# Dysfunction of Microcirculation in Atherosclerosis: Implications of Nitric Oxide, Oxidative Stress, and Inflammation

**DOI:** 10.3390/ijms26136467

**Published:** 2025-07-04

**Authors:** Marta Aleksandrowicz, Marek Konop, Mateusz Rybka, Łukasz Mazurek, Monika Stradczuk-Mazurek, Mateusz Kciuk, Bożena Bądzyńska, Leszek Dobrowolski, Marta Kuczeriszka

**Affiliations:** 1 Laboratory of Preclinical Research and Environmental Agents, Mossakowski Medical Research Institute, Polish Academy of Sciences, 5 A. Pawińskiego Street, 02-106 Warsaw, Poland; maleksandrowicz@imdik.pan.pl; 2 Laboratory of Centre for Preclinical Research, Department of Experimental Physiology and Pathophysiology, Medical University of Warsaw, 3C Pawińskiego Street, 02-106 Warsaw, Poland; marek.konop@wum.edu.pl (M.K.); mateusz.rybka@wum.edu.pl (M.R.); lukasz.mazurek1@wum.edu.pl (Ł.M.); stradczukmonika@outlook.com (M.S.-M.); 3 Department of Molecular Biotechnology and Genetics, Faculty of Biology and Environmental Protection, University of Lodz, 12/16 Banacha Street, 90-237 Lodz, Poland; mateusz.kciuk@biol.uni.lodz.pl; 4 Department of Renal and Body Fluid Physiology, Mossakowski Medical Research Institute, Polish Academy of Sciences, 5 A. Pawińskiego Street, 02-106 Warsaw, Poland; bbadzynska@imdik.pan.pl (B.B.); lesdobro@imdik.pan.pl (L.D.)

**Keywords:** atherosclerosis, endothelial dysfunction, inflammation, microcirculation, oxidative stress, hypercholesterolemia, hyperglycaemia

## Abstract

Cardiovascular diseases (CVDs) are the leading causes of death worldwide, and most of them are connected with atherosclerosis (AS). Hypertension (HT), hyperlipidemia (HPL), and hyperglycaemia (HG) are the main risk factors responsible for CVD and have become a significant public health issue. AS might be a prime causative factor in CVD, and it originates from endothelial cell dysfunction. On the other hand, the factors mentioned above might cause endothelial cell damage as a consequence of endothelial dysfunction (ED) or might be regarded as a consequence of ED. Thus, endothelial cells are critical for maintaining vascular health and homeostasis, and their function is a key contributor to the initiation and progression of AS. The autoregulation of microcirculation, which is functionally present in the brain and kidneys, and from the physiological and pathophysiological point of view, is of high importance to preserve the proper function of the endothelium of blood vessels. The key factor responsible for cardiovascular system regulation and proper action is nitric oxide (*NO*). Disturbances in *NO* synthesis and/or bioavailability, caused by oxidative stress and/or inflammation, accompany or even precede diseases such as HT, angiogenesis-associated disorders, HPL, and HG, which are on the pathway of AS development. In the present review, we attempted to synthesize recent advances in understanding the pathophysiology of multifactorial-related AS.

## 1. Introduction

According to data presented by the World Health Organisation (WHO), hypertension (HT), hyperlipidemia (HPL), and hyperglycaemia (HG) are the main risk factors responsible for cardiovascular diseases and are leading causes of death worldwide, and all of them are connected with atherosclerosis (AS). All of them might be considered cardiovascular diseases (CVDs), which prevail as leading causes of death worldwide and have become a significant public health issue [1]. It is worth indicating that AS might be a prime causative factor in cardiovascular disease, and it originates from endothelial cell dysfunction. In addition, the above factors might cause endothelial cell damage as a consequence of endothelial dysfunction (ED) or might be regarded as a consequence of ED. Endothelial cells (ECs) are critical for maintaining vascular health and homeostasis, and their function is a key contributor to the initiation and progression of AS [1].

ED is regarded as one of the factors responsible for the formation of atherosclerotic plaque and, as a consequence, AS development [2]. The brain and kidneys are regarded as organs of high importance, due to the fact that both possess a very specific blood flow regulation system, and the autoregulation of microcirculation is functionally present in these organs; here we will focus mostly on the brain vasculature. 

The key factor responsible for cardiovascular system regulation and proper action is *NO*. Abnormalities in *NO* synthesis and/or bioavailability accompany or even precede diseases such as HT, angiogenesis-associated disorders, AS, and diabetes [3].

After the last 20 years, since Ignarro’s Nobel Prize, *NO* is still one of the most studied molecule. Briefly, after Hadi and Suwaidi (2007), endogenous *NO* is synthesized by the conversion of L-arginine to L-citrulline, by the group of enzymes called *NO* synthases (NOSs), which produce picomolar amounts of *NO* able to act on a very short distance (paracrine, local effect) [4]. Three distinct genes encode NOS isozymes, which catalyse the production of *NO* from L-arginine: neuronal NOS (nNOS or NOS-1), cytokine-inducible NOS (iNOS or NOS-2), and endothelial NOS (eNOS or NOS-3) [3]. The eNOS-dependent *NO* is produced within the endothelium cells after diffusion to the vascular smooth muscle (VSM) activates the enzyme guanylyl cyclase, and via cGMP, induces vasodilation, as a consequence of VSM relaxation.

From the physiological and pathophysiological point of view, it is of high importance to preserve the proper physiological level of *NO*, by preventing its fast over-synthesis (e.g., caused by early diabetes) and rapid metabolism and reactive oxygen species (ROS) production or even overproduction. ROS caused strong negative effects, leading, among others, to the mentioned endothelial dysfunction and, in consequence, AS. AS is primarily regarded as an inflammatory reaction of the cardiovascular system caused by endothelial damage. Microcirculation, comprising arterioles, capillaries, and venules with a diameter smaller than 20 µm, plays a crucial metabolic role, participating in oxygen delivery, nutrient exchange, and maintaining tissue homeostasis. Microcirculation is most characteristic of the retina, heart, brain, and kidneys, and those organs are the most susceptible to injuries, called microangiopathy. Microangiopathy observed within vessels in the specific vascular beds might lead to life-threatening diseases of these two crucial organs: the brain and kidneys.

The scope of the AS basis is as follows:

1. oxidative stress;
2. endothelial dysfunction;
3. inflammation.

All of these are also responsible for other cardiovascular diseases, which are on the pathway to AS development.

HT induces primary structural changes in the systemic microcirculation: rarefaction, which means a reduction in vessel density, and remodelling, i.e., structural modifications of resistance small vessels, arteries, and arterioles, i.e., microcirculation [5]. In essential HT, remodelling often involves a narrowing of the internal lumen and an increase in the thickness of the tunica media or total/whole vessel wall [5]. HT exerts a profound impact on the microcirculation, causing both structural and functional alterations that contribute to systemic and organ-specific vascular damage. As it was mentioned by Hadi and Suwaidi (2007), endothelial dysfunction has been demonstrated in patients with HT, which is one of the features of the insulin resistance syndrome [4].

Diabetes is a heterogeneous group of diseases characterized by hyperglycaemia. Despite advances in care, patients with diabetes have a two- to fourfold increased risk for developing cardiovascular disease compared with individuals who do not have diabetes and are in a high-risk group.

As described by Derosa and Maffioli (2016), several epidemiological studies showed an evident linkage between diabetes and cardiovascular events [6]. As mentioned, hyperglycaemia enhances the secretion of vasoconstricting factor, endothelin-1 (ET-1), and decreases *NO* production in the aorta of diabetic rats and coronary microvessels in humans [6]. Of note, according to Hadi and Suwaidi (2007), acute experimental hyperglycaemia is responsible for significantly increased plasma *NO* levels, more in subjects with diabetic glucose tolerance than in subjects with normal and impaired glucose tolerance at baseline [4].

In the context of hyperglycaemia, the suppression of endothelial *NO* (synthesized via eNOS) production leads to microcirculation AS, heightened inflammation, and abnormal intimal growth. Hyperglycaemia causes peripheral vascular changes that result in ED and decreased vasodilator secretion, leading to ischemia [7]. Endothelial dysfunction has been demonstrated in insulin-resistant states in both animals and humans and might be considered as a first step to AS development [4].

AS is a complex pathologic process involving various cellular and molecular events, and ED has been regarded as a critical and initiating factor in the pathogenesis of AS [1,8]. Also, there is an increasing amount of evidence that suggests hyperinsulinemia is linked with the development of AS in patients with diabetes.

As mentioned by Shao and coworkers (2024), AS is caused by an inflammatory response resulting from damage to the cardiovascular endothelium, leading to the progressive thickening and hardening of the vascular wall [2]. Within this process is observed a developing and progressing in the presence of risk factors, including hyperlipidaemia, hypercholesterolemia, and chronic inflammation, among others [9]. The pro-atherogenic stimuli causing endothelial dysfunction, next to the mentioned inflammation, include hypercholesterolemia, oxidative stress (OS), HT, metabolic disorder, sex hormone dysregulation, aging, and haemodynamic forces [1,10].

Mounting evidence indicates that ED serves as an initial trigger and pivotal step in the development of AS [10,11]. As mentioned, ED is considered a hallmark of AS and might be treated as a target for AS prevention and management [1].

ECs form a precise barrier between the blood vessel wall and blood, performing numerous essential functions [11]. ECs play a crucial role in maintaining vascular homeostasis, optimizing redox balance, and regulating inflammatory responses. ECD has a critical role in the pathophysiology of AS (in generating the atherosclerotic plaque/lesion), among others, by promoting the upregulation of adhesion molecules, enhanced LDL (low-density lipoprotein) oxidation, platelet activation, and VSM cell (VSMC) proliferation and migration [12]. A healthy endothelium inhibits platelet and leukocyte adhesion to the vascular surface and maintains a balance of profibrinolytic and prothrombotic activity [4]. As was stated by Hadi and Suwaidi (2007), the endothelium is a complex organ, with paracrine and autocrine function, which is in fact a “first line” physiological defence against AS [4].

Sies and coworkers (2017), for the first time, described the imbalance between oxidant production and antioxidant defence as an “OS” [13]. ECs produce mediators that might induce vasoconstriction (e.g., endothelin, prostaglandins, and angiotensin II) and vasodilators like *NO*, which is the most famous one. Endothelium-dependent vasodilation might be assessed in both coronary and peripheral circulation, mostly microcirculation.

To make the OS easier to understand, it is worth knowing that mammalian bodies are permanently producing a large number of free radicals, on a daily basis. If free radicals are not neutralized by any antioxidant, this might lead to the situation of the mentioned OS.

In the context of hyperglycaemia, the suppression of endothelial *NO* production by NOS inhibition leads to an elevated level of ROS, notably superoxide radicals. A key consequence of OS is the reduction in the availability of *NO*, a critical molecule for maintaining vascular homeostasis through vasodilation [5].

OS negatively affects multiple biochemical pathways. Free radicals are generated as by-products of normal metabolic processes, primarily through mitochondrial respiration and energy production, leading to the formation of ROS such as superoxide anions and hydrogen peroxide. It was estimated that the maintenance of vascular function by endothelial *NO* is essential under physiological conditions. Although, in blood vessel walls, *NO* is mainly produced from L-arginine by eNOS, other mechanisms of vascular *NO* production exist. L-arginine was the first discovered and best-characterized source of *NO* as the substrate for NOS [3].

## 2. Factors Involved in Endothelial Dysfunction in Atherosclerosis

A key factor in the development of AS is ED. This dysfunction not only promotes plaque formation in larger arteries but also impairs the ability of the microcirculation to dilate and constrict properly, leading to further impairments in blood flow regulation. ED is predominantly characterized by the secretion of vasoconstrictors such as ET-1 and thromboxane A_2_ rather than vasodilators like *NO* and prostacyclin (PGI_2_) [10,14]. The main factor of endothelial dysfunction is OS, which is linked to elevated ROS concentration [15]. According to Cassuto et al. (2014), one of these, superoxide anion (O_2_^−^), combines with *NO* to form the aggressive nitrogen free radical peroxynitrite [16]. This, in turn, damages the endothelium caveolae, resulting in the uncoupling of endothelial *NO* synthase (eNOS) and a decrease in *NO* bioavailability. In addition to OS, inflammation is also an important factor in the development of ED [17]. In the microvessels, the cytokines such as IL-1β and IL-6 might promote the development of inducible NOS (iNOS) [18]. In healthy vascular tissue, inducible NOS is typically undetectable, but when it is produced, it generates toxically high levels of *NO*, which causes eNOS uncoupling [19]. TNF-α is another strong inducer of iNOS activation in endothelial cells [20]. In microvessels, TNF-α has been shown to be a strong inhibitor of *NO*-mediated endothelium-dependent relaxation [21]. Additionally, eNOS mRNA levels in endothelial cells are directly decreased by high TNF-α concentrations [22,23].

**Figure 1:**
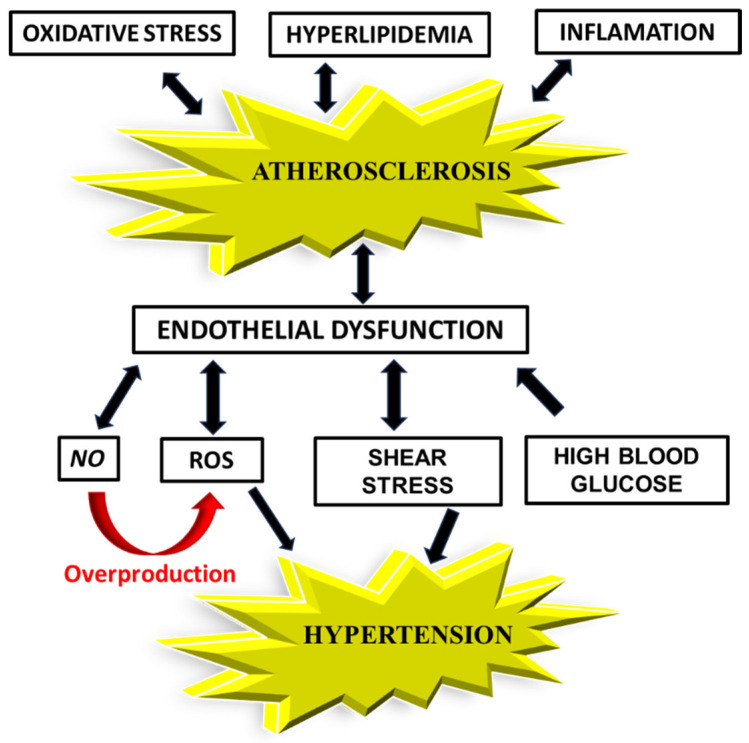
Schematic pathway of the relationship between the factors affecting atherosclerosis, endothelial dysfunction, and hypertension. *NO*—nitric oxide, ROS—reactive oxygen species.

Elevated glucose levels, dyslipidemia, and other metabolic alterations are involved in the pathogenesis of AS. In this section, we discuss their contribution, in addition to OS and inflammation, to endothelial dysfunction in AS. Together, these elements contribute to a cycle of vascular injury that underpins the development of atherosclerotic disease (Figure 1).

### 2.1. Oxidative Stress

The cause of OS is an imbalance between the generation of oxidants and their scavengers [15]. One of the main indicators of OS and a major contributor to the development of AS is the production of ROS. Increased ROS levels, especially from oxidized LDL (ox-LDL), are a crucial early event in AS and contribute to ED. Some experimental studies have demonstrated an increase in the production of superoxide anion in blood vessels in AS [24,25]. Furthermore, superoxide anion binds *NO* in a reaction that produces an aggressive nitrogen free radical, peroxynitrite (ONOO^−^). The disruption of endothelial caveolae by peroxynitrite results in the uncoupling of eNOS and a decrease in *NO* bioavailability [16]. As expected, vascular nitrosative stress was reported in animal models of AS [26]. Simultaneously, high-fat diets reduce the expression of genes involved in free radical scavenging [27], which additionally contributes to the imbalance between the oxidants and their scavengers. For example, Zhang and coworkers (2014) showed decreased activity of superoxide dismutase (SOD), an antioxidant enzyme, in cerebral microvessels in atherosclerotic groups of animals [28].

Decreased *NO* levels cause vasoconstriction [29]. Enhanced production of vasoconstricting ET-1 [30,31] and decreased production of vasodilating *NO* [32,33] have often been described in AS. For example, Fan with colleagues (2000) showed that ET-1 and ET receptors are upregulated in both human and experimental animal atherosclerotic lesions. Moreover, plasma ET-1 levels were significantly elevated in hypercholesterolemic subjects and cholesterol-fed animals [31]. Deficiency in *NO* leads to impaired endothelium-dependent relaxation in vessels of atherosclerotic patients and hypercholesterolemic animal models [26].

Deficiency in the *NO* pathway due to vascular OS also contributes to the progression of AS by facilitating smooth muscle cell proliferation and inflammatory processes [34,35]. ED and plaque instability result from OS impact on the expression of several genes linked to inflammatory responses, such as adhesion molecules and chemokines [36,37]. Inflammation-induced OS results, in turn, in an increased accumulation of ROS, mainly derived from mitochondria [37]. High OS has also been shown to be associated with increased lipid build-up and macrophage infiltration in artery walls, which accelerates atherogenesis [38,39]. Conditions such as hypercholesterolemia, where lipid metabolism abnormalities dramatically increase OS levels, additionally aggravate this process [40,41].

Notably, therapies aimed at reducing OS have shown promise in mitigating the effects of AS. Restoring the equilibrium between ROS and the body’s antioxidant defences can be greatly aided by antioxidants. Antioxidant treatments may prevent AS by lowering ROS levels, enhancing endothelial function, and lowering inflammatory reactions [29]. For instance, compounds like curcumin and fisetin have been studied for their ability to lower OS, suggesting they could be helpful additional treatments for AS [36,38].

Statins, aside from lowering cholesterol, present some supplementary non-lipid effects, i.e., pleiotropic effects. They also boost eNOS activity, leading to increased *NO* synthesis, but not always its bioavailibilty; which might be reduced by increased ROS synthesis. Recently, it was reported in studies using animal models that the beneficial effect of statins could be based on the improvement of e-NOS expression and *NO* production in the vasculature [42,43].

They can also attenuate downstream deleterious effects of factors like protein Rac1 or nicotinamide adenine dinucleotide phosphate (NADPH) oxidase, involved in the production of ROS, which can contribute to ED, inflammation, and oxidation of LDL particles, which all contribute to the development of AS [44,45]. This may partially explain how statins mediate oxidative stress and inhibit AS. 

On the other hand, Krueppel-like factors (KLFs), essential transcription factors in eukaryotes, were reported to be induced by statins (mevastatin, simvastatin, and lovastatin). They were found to induce KLF2 expression, while reduced KLF2 expression attenuates the statin-mediated accumulation of eNOS and platelet-derived growth factor levels [46].

As reported by Sikora et al., the next effect of statins is the reduction in platelet aggregation and the exertion of antithrombotic action [47]. Reduction in oxidative stress and anti-inflammatory properties are two other important pleiotropic traits of statins. They are responsible for the suppression of the formation of ROS and the emission of proinflammatory cytokines [48].

Going further with the pleiotropic properties of statins, they stabilize the plaque and amend the composition of atheroma by decreasing non-calcified plaque and increasing the fraction of the density of calcified plaque. Several clinical trials using the intravenous ultrasound technique confirmed their plaque stabilization action [48].

Next to statins, natural flavonoids (plant-derived phenolic compounds) are in the spotlight of attention as potential drugs with endothelium-protective properties. They act mostly through regulating the endothelial function, targeting inflammation, ROS, impaired *NO* synthesis, and apoptosis. More details can be found in the recent review of Zhong and coworkers (2025) [1].

### 2.2. Inflammation

Inflammation is a complex biological process influenced by multiple factors and involving various cell types, such as endothelial cells, VSMCs, and immune cells like neutrophils, monocytes/macrophages, and lymphocytes, along with mediators such as cytokines, chemokines, and ROS [49,50]. In general, this process aims to remove or destroy the cause of cellular damage and eliminate any cells or necrotic debris formed due to the damage [49]. From a clinical point of view, the inflammation can be acute or chronic. Acute inflammation represents an immediate systemic response to recruit leukocytes to the injury site to eliminate the causative agent. It is characterized by a short duration, the presence of an exudate, and a predominant neutrophilic infiltrate [49]. On the other hand, chronic inflammation is associated with many cells, including monocytes, monocyte-derived macrophages, dendritic cells, and T cells [49,51,52]. Macrophages are the most important listed cell types characterizing chronic inflammation because they secrete many biologically active substances [52].

There is strong evidence that chronic inflammation increases the risk of cardiovascular disease [53]. Regardless of the severity of the inflammation, if it lasts for a long time, it increases the risk of AS, regardless of the primary disease. This is what happens in periodontitis, which increases the risk of myocardial infarction and stroke [54]. This is most likely due to the presence of bacteria in the blood that stimulate the immune system to produce inflammatory cytokines, thus causing vascular damage. A similar situation can be observed in transplanted organs, where inflammation and vascular damage are the main factors responsible for acute and chronic rejection [55]. On the other hand, there is no evidence suggesting that acute inflammation, typically associated with infections, increases the risk of atherosclerotic disease. The formation of ROS is one of the key components of the inflammatory response. In the vascular system, they are produced by cells such as VSMCs and endothelial cells [56]. Unfortunately, free radicals also react with LDL, leading to the production of ox-LDL, directly involved in atherogenesis. In addition, VSMCs react to higher levels of ox-LDL. These cells start to produce proinflammatory cytokines. This creates a cycle in which the problem gets worse over time [57].

AS is a long-term inflammatory condition marked by ED and changes in blood vessel structure [58]. It starts with the activation of ECs prompted by proinflammatory factors, including ox-LDL and irregular blood flow patterns. This activation increases adhesion molecules like vascular cell adhesion molecule-1 (VCAM-1) and intracellular adhesion molecule-1 (ICAM-1) on the endothelial surface [58,59,60]. These molecules facilitate the adhesion and migration of monocytes into the intima, where they transform into macrophages and consume modified lipids, forming foam cells [50]. These factors interact via multiple receptors, for example, toll-like receptors (TLRs), and NOD-like receptors on immune and vascular cells, that activate proinflammatory signalling pathways such as the nuclear factor kappa-light-chain-enhancer of activated B cell (NF-κB) transcriptional cascade [61].

NF-κB increases the likelihood of atherosclerotic plaque rupture by increasing metalloproteinase (MMP) synthesis [14,61]. Additionally, proteins in this pathway directly affect the release of proinflammatory cytokines, such as IL-6 and IL-1β. Studies on mice have shown that IL-6 is involved in remodelling the extracellular matrix and atherosclerotic plaque. Elevated blood levels of IL-6 are associated with poor cardiovascular prognosis and overall mortality in humans, regardless of race or ethnic group [62]. Despite the evident role of inflammation in AS, commonly used anti-inflammatory drugs (e.g., NSAIDs and steroids) do not protect the cardiovascular system. This is most likely due to the inhibition of prostacyclin production and the mineralocorticoid activity of glucocorticosteroids, which leads to increased blood pressure [63,64]. A meta-analysis of 14 observational studies showed that tocilizumab (an IL-6 receptor inhibitor) reduces the risk of major adverse cardiovascular events in patients with severe rheumatoid arthritis [65]. Similar results were obtained for canakinumab, a drug that blocks the IL-1β receptor. Unfortunately, the usefulness of these immunosuppressive drugs is limited because they increase the risk of opportunistic infections and cancer.

However, it is worth mentioning that, while statins are not typically anti-inflammatory drugs, they also exhibit an important anti-inflammatory component in their pleiotropic action [66]. Older drugs such as colchicine, which has an anti-inflammatory effect by inhibiting the activation of the NLRP3 inflammasome, have an FDA-approved role in the secondary prevention of coronary artery disease [67]. Additionally, new strategies using nanoparticle-mediated drug delivery systems are attracting a lot of attention, as they can deliver anti-inflammatory agents directly to inflamed plaques, potentially reducing the risk of adverse effects [68].

### 2.3. NO and Shear Stress

Vascular endothelial cells are continuously exposed to the unique environment created by pulsatile blood flow, where shear stress, the tangential force exerted by flowing blood on the vessel’s inner wall, plays a significant role. This shear stress increases proportionally with the volume of blood flow and is inversely proportional to the diameter of the blood vessel. Changes in vessel diameter thus directly affect the magnitude of shear stress experienced by the endothelium [69]. There are a few kinds of shear stress that differ in their impact on the vessels. Disturbed shear stress (low, turbulent, or oscillatory, occurring in vascular areas like branches, curvatures, or bifurcations) increases the expression and activity of proinflammatory, proapoptotic, vasoconstrictor, and oxidant factors in endothelial cells, leading to atherosclerotic plaque formation [70]. Disturbed shear stress upregulates the expression of adhesion molecules such as vascular cell adhesion molecule 1 (VCAM-1) and intercellular adhesion molecule 1 (ICAM-1), facilitating leukocyte adhesion and transmigration into the vessel wall [71,72]. This proinflammatory environment is further amplified by increased secretion of chemokines like monocyte chemoattractant protein-1 (MCP-1) and activation of transcription factors including NF-κB and activator protein 1 (AP-1), which drive the production of cytokines such as tumour necrosis factor α (TNF-α) and IL-6 [71,73]. Concurrently, disturbed shear stress promotes OS through enhanced activity of nicotinamide adenine dinucleotide phosphate (NADPH) oxidases (Nox4, gp91phox), leading to elevated ROS generation and endothelial damage. The suppression of protective factors like KLF2 (Krüppel-like factor 2) and Nrf2 (nuclear factor erythroid 2-related factor 2) under these conditions impairs antioxidant defences, exacerbating oxidative injury and lipid oxidation [70]. In contrast, laminar and high shear stress induce atheroprotective signalling, thereby maintaining endothelial homeostasis and suppressing inflammation [74].

*NO*, synthesized by *NO* synthase (NOS), is a key signalling molecule that exerts complex, often dichotomous, effects on inflammation. The inducible form (iNOS), upregulated by proinflammatory cytokines and microbial components, produces sustained high levels of *NO*, which can promote vascular permeability, leukocyte adhesion, and cytokine release [75]. Elevated *NO* levels enhance the production of TNF-α, IL-6, and IL-1β by immune and endothelial cells, contributing to the amplification of inflammatory responses [76,77]. In ED, *NO* facilitates neutrophil transmigration and promotes OS through peroxynitrite formation and mitochondrial impairment, particularly under pathological conditions such as septic shock and AS [77]. However, *NO* also exhibits anti-inflammatory properties by inhibiting NF-κB activation, suppressing adhesion molecule expression, and promoting regulatory T cell (Treg) differentiation [75]. In cutaneous and neuroinflammatory models, *NO* donors reduced inflammatory infiltration, cytokine release, and disease severity, partly via modulation of inflammasome activity and T cell apoptosis [76,78]. These effects are context-dependent: low physiological *NO* concentrations maintain vascular homeostasis, while excessive or prolonged *NO* production can be cytotoxic and immunosuppressive [79]. Thus, the dual role of *NO* in inflammation reflects a delicate balance of concentration, NOS isoform activity, and local cellular context, positioning *NO* as both a driver and a regulator of inflammatory pathways.

### 2.4. Hypercholesterolemia

Hypercholesterolemia is a lipid disorder in which the level of LDL is increased in the blood [80,81]. The relationship between LDL and AS is supported by numerous cellular and biochemical mechanisms that lead to plaque formation [82,83]. The main mechanism by which LDL is involved in the development of AS is its oxidation because ox-LDL is particularly atherogenic, as it is readily taken up by macrophages via scavenger receptors such as CD36. It leads, in turn, to the formation of foam cells [84]. Additionally, ox-LDL contributes to a vicious cycle of inflammation by causing endothelial cell dysfunction, which facilitates leukocytes and other immune cells to infiltrate the artery wall [84,85].

Hypercholesterolemia is a main risk factor for coronary and peripheral vascular diseases [80,86]. However, there is increasing evidence that hypercholesterolemia also leads to microvascular dysfunction long before the appearance of atherosclerotic lesions in large vessels [81]. Hypercholesterolemia-induced microvascular dysfunction manifests itself in arterioles as impaired endothelium-dependent vasodilation, as well as leukocyte and platelet migration [81]. Endothelial activation and increased levels of proinflammatory cytokines contribute to microvascular changes in hypercholesterolemia. These events may ultimately lead to thrombosis [87]. Indeed, Kraft et al. (2017) demonstrated erythrocyte stasis and thrombotic occlusions of microvessels in hypercholesterolemic animals [88]. Additionally, hypercholesterolemia causes increased expression of endothelial proteins, including P-selectin and adhesion molecules [86,89], increased blood viscosity [90], and autoregulatory changes [91], which consequently cause an inflammatory/thrombotic state in the microvessels. Long-term increases in LDL induce plaque formation in cerebral microvasculature [92]. Hypercholesterolemia also leads to OS in cerebral arterioles, which in turn, causes ED in these vessels [93].

Despite the adverse effects of hypercholesterolemia on cerebral microvessels, the relationship between high cholesterol and ischemic stroke is not obvious. Some studies indicate a close relationship between cholesterol levels and ischemic stroke [94,95,96]. On the other hand, other studies have shown no correlation between them [97,98,99]. Clinical studies have demonstrated that, for example, type 2 diabetes or HT poses a greater risk of cerebral small vessel disease compared to hypercholesterolemia [92]. This discrepancy could be associated with stroke subtypes, sex, and the age of patients [98,100]. Furthermore, cerebral vessels differ from other vascular beds with respect to endothelial cell function and resistance to injuries relative to coronary vessels or other peripheral vessels [101].

Moreover, no positive relationship between cholesterol and stroke mortality, especially at older ages (70–89 years vs. 40–59 years of age) or higher blood pressures, has been explained, and the topic needs further research [98].

Interestingly, despite the inconsistent association between cholesterol and stroke, statins, which lower cholesterol, reduced the risk of ischemic stroke [102,103]. This could be related to the beneficial effects of statins on endothelial function, as well as the anti-inflammatory and antithrombotic effects of the drugs [93,102].

### 2.5. Hyperglycaemia

Hyperglycaemia is defined as a state in which blood glucose levels exceed physiological ranges. The most common cause of hyperglycaemia is *diabetes mellitus*, in which there is impaired insulin production (type 1 diabetes) or resistance to the peripheral actions of insulin (type 2 diabetes). Type 1 and type 2 diabetes are significant risk factors for coronary artery disease and stroke [104]. Diabetes is a multifactorial condition, and in addition to hyperglycaemia, it is linked to abnormal glucose fluctuations, lipid changes, hormone changes, and a proinflammatory state [105].

Hyperglycaemia causes undesirable changes in vascular tissue that, in turn, accelerate the atherosclerotic process [93]. Its effect on blood vessels is mainly related to inflammatory processes and OS, which together contribute to the pathogenesis of AS. Hyperglycemia is associated with increased inflammation in cerebral microvessels [106]. Nishizawa et al. (2014) showed that high glucose conditions cause greater macrophage accumulation in the vessel wall [107]. Furthermore, Nagareddy et al. (2013) found that hyperglycaemia promotes myelopoiesis, leading to enhanced numbers of proinflammatory monocytes [108]. Elevated glucose leads to increased release of cytokines and chemokines from macrophages [105,106]. Moreover, Rom et al. (2019) showed an increase in gene expression of adhesion molecules in cerebral microvessels [106].

In addition to inflammation, high glucose levels also increase OS. An increased superoxide level was reported in cerebral parenchymal arterioles in hyperglycaemia [109]. Zheng et al. (2010) demonstrated that intermittent high glucose exposure can evoke apoptosis of ECs due to mitochondrial superoxide overproduction [110]. Hyperglycaemia can also reduce levels of natural antioxidants [111,112]. The increased expression of genes responsible for the release of free radicals is closely related to the interaction of advanced glycosylation end products (AGEs) and their receptor REGE [104,113]. AGEs are the products of the reaction of monosaccharides, including glucose, with proteins, lipids, and nucleic acids. The interaction of AGEs with their receptors has been shown to evoke inflammatory and thrombotic reactions [114]. The presence of RAGE receptors has been reported in all cells relevant to the atherosclerotic process, including monocyte-derived macrophages and endothelial cells [115,116]. Receptor-mediated mechanisms activate the mechanisms leading to dysfunction of the endothelium through increased expression of adhesion molecules [115], increased intracellular stress [113], and increased procoagulant activity [117].

Dysfunction of the endothelium was reported in cerebral parenchymal arterioles in hyperglycaemia [118]. High concentrations of glucose impair the endothelium-dependent relaxation of parenchymal arterioles [118]. Among other things, the response of parenchymal arterioles to the vasodilators acetylcholine and ADP was impaired [118]. On the other hand, some data showed that hyperglycaemia had no effect on cerebral blood flow and basal tone of parenchymal arterioles [119]. Moreover, a beneficial effect of hyperglycaemia on lacunar stroke was reported [120,121]. This can be explained by the fact that, under conditions in which *NO* release is impaired, another vasodilator, endothelium-dependent hyperpolarizing factor (EDHF), can compensate for the loss of *NO* [119]. Interestingly, it was reported that the poor outcome in acute ischemic stroke (AIS) could not be attributable to the diabetic status. During a review of medical records of patients with AIS, no difference was noted in the outcome between diabetic and nondiabetic patients [122].

However, a meta-analysis of 68 prospective studies (including around 1 million adults) indicated that diabetic patients were inclined to increased vascular mortality, particularly from occlusive causes, including ischaemic stroke, or other atherosclerotic deaths. Moreover, the sex differences analysis indicated that a higher relative risk exists among women than among men. However, what exactly made these differences still remained unexplained. It seemed that they could not be related solely to established major vascular risk factors, such as blood pressure, total cholesterol, body mass index (BMI), and smoking status [123].

**Figure 2:**
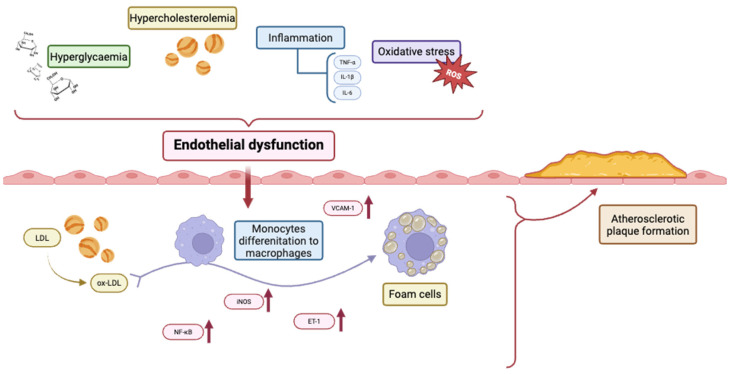
Pathophysiological mechanisms contributing to atherosclerotic plaque formation. “Created in BioRender. Konop, M. (2025) https://BioRender.com/md0y6li” (URL accessed on: 23 June 2025).

The figure below (Figure 2) illustrates the interaction between hyperglycaemia and hypercholesterolemia in inducing endothelial dysfunction through the release of inflammatory cytokines (e.g., TNF-α, IL-1β, and IL-6) and the generation of oxidative stress (reactive oxygen species, ROS). These processes increase vascular permeability and the expression of adhesion molecules such as VCAM-1, aiding the recruitment of monocytes. Low-density lipoproteins (LDLs) undergo oxidative modification (ox-LDL) in the subendothelial space and are absorbed by differentiated macrophages, which turn into foam cells. Activation of NF-κB, inducible nitric oxide synthase (iNOS), and endothelin-1 (ET-1) further heightens inflammation. The build-up of foam cells and unresolved inflammation contributes to the formation and progression of atherosclerotic plaques.

## 3. Cerebral Microcirculation in Atherosclerosis

**Figure 3:**
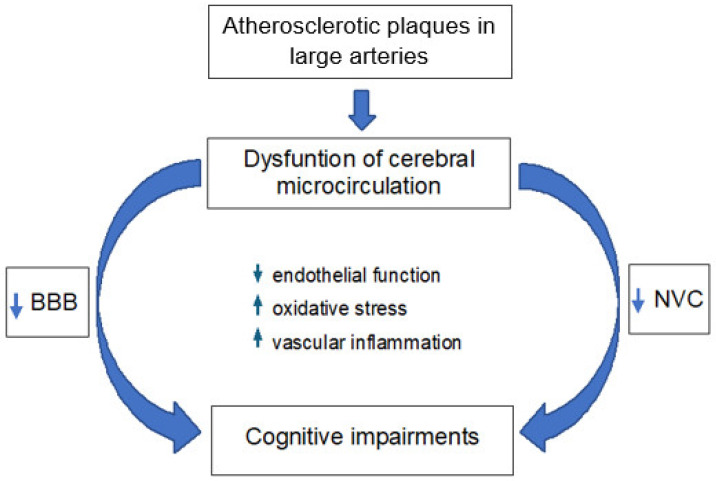
Correlation between dysfunction of microvessels and cognitive impairments. Atherosclerotic plaques in large arteries lead to dysfunction of cerebral microcirculation, which in turn, translates into dysfunction of the blood–brain barrier (BBB) and neurovascular coupling (NVC). Both of these are involved in the development of cognitive impairments and ischemic events. Dysfunction of cerebral microcirculation in atherosclerosis is associated with disordered functions of the endothelium, due to vascular inflammation and oxidative stress.

Although atherosclerotic plaques grow within the large arteries, such as the carotid arteries and large cerebral arteries, they also reduce the overall cerebral blood flow (CBF). This leads to improper perfusion in the downstream microcirculation [124,125], thereby contributing to the development of cerebral small vessel disease (CSVD). Moreover, increased arterial stiffness of large arteries leads to increased pulse-wave velocity and pulse pressure [126,127], which in turn, may influence cerebral small vessels. Microcirculation, defined as the network of parenchymal arterioles, capillaries, and venules, is crucial for maintaining proper cerebral perfusion. Microvessels directly determine CBF through changes in their diameter. Dysfunction of microvessels may result in the impairment of functional hyperemia, also named neurovascular coupling (NVC), and disruption of the blood–brain barrier (BBB). Impairments within both NVC and the BBB may lead to cognitive impairments [128,129] (Figure 3). In the section below, we discuss the influence of AS on NVC, the BBB, and consequently on the occurrence of cognitive disorders.

### 3.1. Neurovascular Coupling

In a normal brain vascular system, vasodilation adjusts the cerebral microflow in response to increased demand for oxygen and nutrients during increased neuronal activity. This NVC is reduced or absent in many forms of cerebrovascular pathology [130]. NVC requires the structural and functional integrity of the neurovascular unit. The neurovascular unit [NVU] is composed of microvascular endothelial cells, astrocytes, neurons, and pericytes, which are interconnected and function in concert. Neuronal activity is communicated to the cerebral vasculature at the level of the neurovascular unit and precapillary arterioles to adjust the local perfusion of brain tissue to neuronal metabolism. An increase in neuronal activity is accompanied by vasodilation and by an increase in local cerebral blood flow. This neurovascular coupling is essential for the homeostasis and proper functioning of the brain, and endothelial cells are an indispensable component of the neurovascular unit, contributing to the regulation of the microflow [131].

Dysfunction of cerebral microcirculation can critically affect neurovascular coupling. Alterations within microvessels, particularly those induced by aging and AS, could lead to neurodegenerative conditions and cognitive dysfunction [132]. It is known that AS affects cerebral microcirculation, contributing to cognitive decline and increased risk of ischemic events [133,134,135]. Some studies demonstrated that disruptions in neurovascular coupling, possibly due to microvascular changes from systemic atherosclerotic factors, significantly impair local CBF regulation. Eyre et al. (2025) demonstrated a disturbed response to whisker stimulation in mice with AS [135]. Lu et al. (2019) found similar results, demonstrating that the somatosensory cortex of aged atherosclerotic (ATX) mice had a reduced hemodynamic response to whisker stimulation [133]. Compared to juvenile atherosclerotic mice, ATX animals also showed a lower and more variable partial pressure of oxygen (pO_2_) in cerebral tissue. Additionally, hypoxic micro-pockets in cortical tissue were found in old, but not young, ATX mice. Another study confirmed that old atherosclerotic mice exhibit a higher spatial heterogeneity of tissue pO_2_, suggesting a less efficient oxygen extraction [134].

The worsened microvascular perfusion contributes to ischemic damage in the cerebral tissues. AS causes unnoticed strokes that occur in areas of the brain with compromised blood flow. Over time, these silent strokes can accumulate, leading to subcortical ischemic changes [136]. Some studies confirmed that atherosclerotic disease plays a causative role in the onset of ischemic cerebrovascular diseases [137,138]. Clinical studies showed that, in patients with AS, lacunar infarcts are observed [139,140]. Interestingly, smaller lacunes (<3 mm) were associated with diabetes, and larger lacunes were associated with high LDL cholesterol [141].

### 3.2. Blood–Brain Barrier

Endothelial cells in the cerebral microvessels form the BBB, which limits the transport of undesirable substances from the bloodstream to the brain. Cerebral endothelial cells are connected by tight junctions (TJs), which are composed of integral membrane proteins such as occludin and claudin-5. Both membrane proteins are linked to the actin cytoskeleton by TJ-associated proteins such as zonula occludens-1 (ZO-1) [142].

The majority of research on the relationship between AS and the BBB structure shows that carotid artery stenosis and AS in large arteries cause disruption of the BBB [143,144]. More precisely, increased BBB permeability and tight junction dysregulation have been reported [124,145,146,147]. Furthermore, increased BBB permeability is linked to AS risk factors like HT, HPL, and HG [148,149]. Changes in the expression and structure of endothelial TJs have been documented in these comorbidities [150,151,152]. Additionally, it has been demonstrated that proinflammatory cytokines impair the endothelial barrier [153,154]. Elevated OS and inflammatory mediators interact to create a vicious cycle that damages the endothelium and increases the BBB’s permeability (Figure 3). Consequently, it facilitates potentially harmful substances to enter the central nervous system, which can lead to neurological conditions and cognitive decline [155,156]. On the other hand, some studies indicated circumscribed BBB breakdown in the white matter in AS [88], which could, however, be an early predictor of white matter damage commonly found in cerebral small vessel diseases [157].

OS and inflammation cause BBB dysfunction, leading to impaired brain function in patients with AS. Understanding the impact of AS on BBB permeability is crucial to developing treatment strategies that will preserve the integrity of the BBB and reduce the incidence of neurodegenerative diseases associated with this systemic disorder [156,158].

### 3.3. Cognitive Impairments

Normal cognitive functions require an adequate, well-regulated delivery of blood to the neurons [159]. The oxygen and glucose are delivered to neurons by microvessels; therefore, microvascular dysfunction may be associated with worse cognitive performance. Accumulating evidence supports the role of disturbance of neurovascular coupling and BBB functions in the pathophysiology of cognitive decline [128,129,160]. Despite disturbed BBB and NVC functions in AS [124,133,134,146,147], conclusions regarding the effect of AS on cognitive function appear inconsistent. The vast majority of studies indicate that AS leads to reduced cognition [140,161,162,163,164]. Cognitive disorders occur even in subclinical AS [165]. On the other hand, a few studies showed no association between markers of AS and cognitive dysfunction [166,167,168]. For example, Auperin et al. (1996) did not identify any statistically significant correlations between AS and cognitive function in women [166]. The discrepancy between studies indicating the existence or absence of a correlation between AS and cognitive disorders may be related to study sample variability, cognitive testing techniques, and AS severity. Additionally, it should be noted that factors that often accompany AS, e.g., older age, can themselves cause the development of cognitive disorders.

In conclusion, the connection between AS and cerebral microcirculation is essential to comprehending the pathogenesis of vascular cognitive impairment (Figure 3). The detrimental effects of AS on cerebral microcirculation may be mitigated by early management and systemic microvascular health monitoring, preserving cognitive function and reducing the risk of dementia.

## 4. Final Remarks

Our review article described the multifactorial pathophysiology of AS, emphasising the key roles of microcirculatory dysfunction, OS, inflammation, and NO bioavailability. Atherosclerosis remains the leading cause of CVDs, which are the leading cause of death worldwide. The endothelium is crucial for vascular health, with its dysfunction serving as both a marker and a mediator of AS progression. This article highlights how hypertension, hyperlipidaemia, and hyperglycaemia impair NO synthesis, increase reactive oxygen species (ROS), and promote chronic inflammation. These mechanisms promote endothelial injury, plaque development, and vascular remodelling. Special attention is given to cerebral microcirculation, where AS impairs neurovascular coupling and BBB function, which may lead to cognitive decline. Hypercholesterolemia and hyperglycaemia further impair endothelial dysfunction through ROS overproduction and advanced glycation end products. This article also discusses disturbed shear stress and proinflammatory cytokines such as TNF-α and IL-6, which disrupt eNOS regulation and intensify inflammatory cascades. Experimental and clinical evidence support the notion that maintaining NO levels and reducing oxidative and inflammatory triggers may help lessen atherosclerosis. Future research should focus on identifying specific molecular markers and NO-modulating pathways to improve targeted therapies for atherosclerosis. Advanced diagnostics and antioxidant-based interventions could allow earlier prevention of AS-related microvascular and cognitive complications.
